# Addressing the language needs of industry: Incorporating real-life workplace communication into SGC-based vocational English modules

**DOI:** 10.1186/2193-1801-3-S1-O6

**Published:** 2014-12-04

**Authors:** Annie Lai-kun Choi

**Affiliations:** Languages Planning and Development Office, Vocational Training Council, Kragujevac, Hong Kong

## Background

For vocational English courses to adequately address the language needs of today’s industries, it is of vital importance to incorporate into the course materials and classroom activities details and characteristics of the real-world communication tasks that trade practitioners are required to perform in their workplaces [1]. As part of a recent study conducted by the Languages Discipline entitled the *Industry Language Training Needs (ILTN) Study*, this paper examines how authentic workplace communicative events derived from the qualitative data collected directly shed light on the language needs of industry, and it explores ways of translating the findings into practical pedagogical uses for the existing SGC-based Vocational English modules of the various programs offered by the Vocational Training Council (VTC).

## Methods

The industry sectors involved in the study include catering, communication design, customer services, hotel, human resources, leisure and sports, merchandising, retailing, sales and marketing, tourism, etc. This paper focusses on an analysis of the data collected through semi-structured interviews with industry practitioners and workplace observations, together with the authentic trade texts obtained from the industries.

## Results

Drawing on a synthesis of Halliday’s [2] triad construct of context of situation in systemic functional linguistics and Douglas’ [3] construct of discourse domain as a theoretical framework alongside the Specification of Generic (Foundation) Competencies (English) (SGC) [4], the analysis found that the numerous specific communicative events (when studied in conjunction with the contextual details) derived from the data, such as replying to email/telephone enquiries, handling complaints, writing reports and proposals, taking part in discussions and meetings/negotiations, giving presentations, etc., are of various levels of complexity and familiarity and require language output of different levels of sophistication. Upon systematically mapping these real-life workplace communication tasks with the corresponding units of competency (UoCs) in the SGC according to the language skills/competency elements involved and the QF levels into which these tasks fit, the majority of these communicative events were found to cluster around those UoCs that are covered in the English curricula of the various VTC programs. This lends substantial empirical support for the relevance of these English modules to the generic language competencies needed in the actual workplace.

Examples from the analyzed data will be used to illustrate how details of these real-life workplace communicative events, especially contextual dimensions including the subject matters, participants/interlocutors and modes of communication that essentially determine language use, can be translated into actual pedagogical tasks for vocational language training, particularly within a framework of outcomes-based teaching and learning[5], and how this facilitates the constructive alignment between the intended learning outcomes, teaching and learning activities and assessment tasks of the existing SGC-based vocational English modules.

## Conclusions

The paper concludes by recommending that a resource bank of authentic workplace communicative events be developed as a practical means of support for vocational English teaching and curriculum development, pointing towards more research-led professional practice for vocational language training.
